# The Relationship Between Patients' Overall ICU Experiences, Psychological Distress, and Sleep Quality Among Jordanian Burn Patients: A Cross-Sectional Study

**DOI:** 10.7759/cureus.54908

**Published:** 2024-02-26

**Authors:** Suhair Al-Ghabeesh, Mohammad Alnaeem, Ahmad Rayan

**Affiliations:** 1 Nursing, Al-Zaytoonah University of Jordan, Amman, JOR; 2 School of Nursing, Al-Zaytoonah University of Jordan, Amman, JOR; 3 Psychiatry, Zarqa University, Zarqa, JOR

**Keywords:** psychosocial stress, burn trauma, sleep quality, psychological distress, icu experience

## Abstract

Objectives

This study aimed to assess patients’ experiences, psychological distress, and sleep quality among Jordanian burn patients.

Method

A cross-sectional, correlational design was used. A sample of 150 patients admitted to the burn-specific intensive care unit completed the study. Demographical data, Intensive Care Experience Questionnaire, and sleep ICU questionnaire were used for data collection. Descriptive and inferential statistics were used for analysis.

Results

Participants were found to have poor sleep quality and severe psychological distress. Sleep quality was negatively associated with awareness of ICU experience (*r = *-.190, *P = *.020) and psychological distress (*r = *-.190, *P = *.020) and positively associated with the recall of experience in ICU. Female participants had significantly greater ICU experience frightening and recall. Participants who experienced burn complications had significantly higher psychological distress.

Discussion

There is a need to offer an encouraging environment to burn patients to improve the psychological health and sleep quality in critical care units.

## Introduction

Burn is a complex traumatic global community health event, responsible for 180,000 deaths yearly [1]. Burn not only affects the skin but also has a systemic effect that leads to disfigurement and dysfunction [2]. A painful treatment of burn injuries during the recovery process could negatively impact patients’ psychological status and delay recovery and wound healing [3]. Psychological distress is the primary complaint after a patient's burn event [4, 5]. Shepherd et al. reported that a high level of anxiety could cause cognitive dispersion among burn patients [6]. Therefore, psychological support is essential to this group of patients [4, 6-7].

Additionally, it is well known that hospitalization affects the sleep quality of patients [8]. Energy is a very important thing to ill patients and can improve their health, and patients can increase their energy through sleep [9]. Protein synthesis and cell division occur during sleep, resulting in a curative process [10]. Patients in ICUs have difficulty falling asleep, and experience reduced rapid eye movement, sleep, and variations in their circadian rhythms due to noise, pain, frequent treatments, and nursing care activities [8, 11]. Sleep deficiency can disturb many organ tasks and decrease immunity, causing cardiovascular abnormalities, cognitive impairment, falling, and bone fracture, especially in the elderly [8]. More than two-thirds of burns frequently happen in patients living in low-income and low-middle-income countries [1]. Non-fatal burns are the remaining causes of sickness and extended hospitalization, depending on the burn's severity [1]. The goal of burn treatment is to be discharged from the hospital with optimal function and be socially active [3]. However, no recent studies found a significant association between the burn injury experience and its effect on those patients' psychological consequences and sleep quality. There is a need to understand the link between the experiences of a burn injury and both the level of psychological distress and sleep quality to determine the impact of burn injuries on these important variables and develop relevant interventions. Especially in Jordan, little is known about the overall ICU experiences, psychological distress, and sleep quality of burn patients in ICU burn units.

Moreover, burn ICU patients in Eastern countries may have exceptional distress. The current study was carried out to understand the experiences of adult patients with severe burns during their hospitalization. This study aimed to bridge the knowledge gap regarding the overall ICU experiences, psychological distress, and sleep quality of patients with severe burns during their hospitalization; this was achieved by exploring their responses to valid and reliable questionnaires. Therefore, this study explored the following research questions:

What is the level of patients' ICU experiences, psychological distress, and sleep quality among burn patients?

Are there differences in patients' ICU experiences, psychological distress, and sleep quality based on participants’ demographics?

Is there a relationship between patients' CCU experiences, psychological distress, sleep quality, and participants’ demographics?

## Materials and methods

Design

A cross-sectional correlational study was conducted. For estimating the sample size, the G*Power 3.1.9.7 program was employed to accomplish this task. Hence, using a multiple regression test, the minimal sample size required was 150 subjects (assuming a medium effect size = 0.15, alpha = 0.05, power = 0.80).

Setting and sampling

Data were collected from a convenience sample of 150 patients admitted to the ICU burn unit and still getting burn care in the most prominent military hospital with a specialized, most extensive burn intensive care unit in Jordan. Conscious patients admitted to the burn-specific intensive care unit minimally two nights were approached. Those with any mental illness and sedated patients were excluded from the study.

Ethical consolidation

This study was approved by the Institutional Review Board of Al-Zaytoonah University (reference no: 4/1/2021-2022), written informed consent was obtained, and the purpose of the study was explained to all participants. Patients' confidentiality was protected. All data were available only to the authors.

Instruments and data collection process

In collaboration with the head nurse of the ICU burn unit, the participants who signed the consent form were asked to complete the self-administered questionnaires. One hundred fifty patients contributed between February and December 2022. The finalized questionnaires were coded and saved in a packet. The instrument contained four parts: (i) Participants' socio-demographics and clinical data, (ii) The Intensive Care Experience Questionnaire, (iii) The sleep ICU questionnaire, and (iv) Kessler Psychological Distress Scale.

A socio-demographic and clinical data questionnaire was developed by the researchers of this study and used to collect information about burn patients (gender, age, marital status, employment status, education, burn type, burn etiology, burn degree, complications), and clinical variables (duration of admission in burn ICU, duration of hospitalization out of burn-ICU).

The Intensive Care Experience Questionnaire (ICEQ) is a valid and reliable tool, consisting of 24 items gathered into four subscales: nine items for awareness of the surrounding subscale, six items for frightening experience subscale, five items for recall of experience subscale, and four items for satisfaction with care subscale. All items' quantity levels of agreement on a 5-point Likert scale and the scores for each subscale were totaled up. Furthermore, the sets of each item were divided into three: agreeable, disagreeable, and neither.

The sleep ICU questionnaire used a 10-point Likert scale on 27 items. Two items measured the overall sleep quality, and three items measured the sleep quality on the first day. One indicates 'no sleep' while 10 indicates 'excellent sleep'. Four items measured the degree of daytime sleepiness in the ICU. The remaining items (18 items) measured sleep disruption in the ICU, 1 indicating “no disruption” and 10 indicating “significant disruption”. In the current study, each item of sleep quality and daytime sleepiness was considered into two (poor vs. good) sleep quality. In contrast, each sleep disruption item was grouped into no vs. significant disruption.

Kessler Psychological Distress Scale (K6) is a well-validated and commonly used clinical measure of psychological symptoms. The Cronbach's α value of the scale equals 0.81, and it has excellent discriminant validity [12]. The scale measures the frequency at which respondents experience psychological symptoms in the past days based on a 5-point Likert-type scale ranging from 1 (none of the time) to 5 (all of the time). The total score of the Kessler Psychological Distress Scale ranged from 6-30, with higher scores representing more psychological distress. However, for data analysis, the scoring is transformed to a 5-point Likert-type scale ranging from 1 (none of the time) to 4 (all of the time), with a total score ranging from 6-24. The scores 0-7 indicate low psychological distress, 8-12 indicate moderate psychological distress, and scores 13-24 indicate severe psychological distress [13].

Data analysis

Data were analyzed by SPSS version 26.0 (IBM Corp., Armonk, NY, USA). Descriptive statistics were used to identify the frequency, percentage, mean, and standard deviation of the demographic variables, patients' ICU experiences, psychological distress, and sleep quality of burn patients and the differences in patients' ICU experiences, psychological distress, and sleep quality based on participants' demographics. Pearson product-moment correlation was used to identify the relationship between patients' ICU experiences, psychological distress, sleep quality, and participants' demographics. Statistical significance was set at *p* = 0.05.

## Results

Sample characteristics

A sample of 150 patients participated in the study. Of them, 91 (60.7%) were women. Participants were aged between 18 and 87 years (M = 36.77, SD = 14.06). More than half of the participants were married (n = 84, 56%). Eighty-four participants (56%) had a secondary school level or below, while the remaining 66 (44%) had a diploma level of education or higher. Forty-four percent of the participants do not have children, while the remaining have at least one child. About 51% had a monthly income greater than 500 JD.

The total body surface area (TBSA) burn was 20% among 92% of the sample. The average length of hospitalization in the ICU was 9.99 days (SD = 8.00), ranging from one day to 42 days, while the average length of hospitalization in the burn unit was 8.37 days (SD = 6.72), ranging from one day to two months. More than half of the participants had flam burns. Most burns had occurred in summer (50.7%) and winter (24%). About two-thirds of the participants (67.3%) had a second-degree burn, while 49 (32.7%) had a third-degree burn. Most burn accidents occurred at home (n = 111, 74%) and at work (n = 24, 16%). Burn complications and associated problems (e.g., infection, scarring, and hypovolemic shock) occurred in 39 participants (26%). The vast majority of the participants were treated with dressing (91.3%). All participants reported that burns occurred accidentally. Only 11 participants (7.3%) had a previous history of a burn incident. Some participants had deformities in one or multiple areas of the body as a result of the burn, including facial deformity (n = 22, 14.7%), hand deformity (n = 16, 10.7%), and other deformities (n = 10, 6.7%). For more information see Table 1.

**Table 1:** Sample Characteristics CCU: Critical care unit

	Categories	Frequency	%
Gender	Male	59	39.3
	Female	91	60.7
Educational Level	Secondary School Level And Below	84	56.0
	Diploma Level And Above	66	44.0
Marital Status	Married	84	56.0
	Single	66	44.0
Monthly Income	Less Than 500 JD	77	51.3
	500 Or More	73	48.7
Number Of Children	No Children	66	44.0
	One Or Two Children	38	25.3
	Three Children Or More	46	30.7
Season When Burn Happened	Summer	76	50.7
	Spring	12	8.0
	Winter	36	24.0
	Autumn	26	17.3
Complications Of Burn	Yes	39	26.0
	No	111	74.0
Type Of Treatment	Dressing	137	91.3
	Skin Grafting	13	8.7
Burn Mechanism	Accidental	150	100.0
Previous Burn	Yes	11	7.3
	No	139	92.7
Space Area Of Burn	20-50%	138	92.0
	More Than 50%	12	8.0
Burn-Related Deformity	None	102	68.0
	Facial Deformity	22	14.7
	Hands Deformity	16	10.7
	Other Locations	10	6.7
Type Of Burn	Flame	79	52.7
	Electrical Burn	14	9.3
	Hot Materials	46	30.7
	Other	11	7.3
Highest Degree Of Burn	2nd Degree	101	67.3
	3rd Degree	49	32.7
Location Where The Burn Happened	Home	111	74.0
	Work Place	24	16.0
	Other	15	10.0
	M	SD	Range
Age	36.77	14.06	18-87
Days in CCU	9.99	8.00	1-42
Days Spent In Burn Unit	8.37	6.72	1-60

Patients' ICU experiences, psychological distress, and sleep quality among burn patients

The overall mean score of sleep quality was 34.25 indicating poor sleep quality. The mean score was 34.24 (SD = 17.44) for CCU experience awareness, 12.19 (SD = 4.16) for ICU experience frightening, 15.83 (SD = 5.37) for ICU recall experience, and 9.44 (SD = 2.38) for ICU experience satisfaction with care. The mean score for psychological distress was 18.38 (SD = 5.05), indicating severe psychological distress (see Table 2).

**Table 2:** Patients' ICU experiences, psychological distress, and sleep quality

	Min	Max	Mean	SD
Sleep Quality	2.40	90.00	34.24	17.44
ICU Experience Awareness	9.00	45.00	26.36	9.21
ICU Experience Frightening	6.00	22.00	12.19	4.16
ICU Recall Experience	5.00	25.00	15.83	5.37
ICU Experience Satisfaction	4.00	16.00	9.44	2.38
Psychological Distress	0.00	24.00	18.38	5.05

Differences in patients' ICU experiences, psychological distress, and sleep quality based on participants’ demographics

The independent samples t-test showed that female participants had significantly greater ICU experience frightening (M = 12.74, SD = 4.05) than male participants (M = 11.33, SD = 4.22), P = .043. Similarly, female participants had significantly greater ICU experience recall (M = 16.39, SD = 5.42) than male participants (M = 15.12, SD = 5.25), P = .043. Participants who experienced burn complications had significantly higher psychological distress (M = 20.23, SD = 4.09) than participants who did not experience complications related to burn (M = 17.73, SD = 5.20), P = .08. However, no significant difference was found in patients' ICU experiences, psychological distress, and sleep quality based on education level (P > 0.05). Regarding income, participants with a monthly income of 500JD or more had significantly less frightening ICU experience (M = 11.20, SD = 4.09) than participants with a monthly income less than 500JD (M = 13.12, SD = 4.04), P = .004. Also, participants with a monthly income of 500JD or more had significantly less recall of ICU experience (M = 14.62, SD = 4.79) than participants with a monthly income of less than 500JD (M = 17.10, SD = 5.68), P = .004.

The relationship between patients' CCU experiences, psychological distress, sleep quality, and participants' demographics

Age was negatively associated with satisfaction with care in the ICU (r = -.242, P = .003). The number of days spent in the ICU was positively associated with awareness of ICU experience (r = .380, P < .001) and psychological distress (r = .191, P = .019) and negatively associated with ICU experience frightening (r = -.242, P = .003). The number of days spent in the burn unit after being discharged from CCUs was positively associated with awareness of ICU experience (r = .349, P < .001) and psychological distress (r = .232, P = .004) and negatively associated with ICU experience frightening (r = -.227, P = .005). Psychological distress was positively associated with awareness of ICU experience (r = .478, P < .001) and negatively associated with ICU experience frightening (r = -.359, P < .001) and satisfaction with care in the ICU (r = -.176, P = .031). Finally, sleep quality was negatively associated with awareness of ICU experience (r = -.190, P = .020) and psychological distress (r = -.190, P = .020) and positively associated with the recall of experience in ICU (r = .208, P = .011) (see Table 3).

**Table 3:** The relationship between patients' ICU experiences, psychological distress, sleep quality, and participants demographics

		ICU Experience Awareness	ICU Experience Frightening	ICU Experience Recall	ICU Experience Satisfaction	Psychological Distress
Age	r	-.098	-.126	.000	-.242^**^	-.033
	P	.232	.125	.997	.003	.685
Days Spent In CCU	r	.380^**^	-.191^*^	-.135	-.101	.191^*^
	P	.000	.019	.099	.219	.019
Days Spent In Burn Unit	r	.349^**^	-.227^**^	.104	-.121	.232^**^
	P	.000	.005	.204	.139	.004
CCU Experience Awareness	r	1	-.199^*^	.213^**^	.001	.487^**^
	P		.014	.009	.986	.000
CCU Experience Frightening	r	-.199^*^	1	-.226^**^	.274^**^	-.359^**^
	P	.014		.005	.001	.000
CCU Experience Recall	r	.213^**^	-.226^**^	1	.253^**^	.060
	P	.009	.005		.002	.464
CCU Experience Satisfaction	r	.001	.274^**^	.253^**^	1	-.176^*^
	P	.986	.001	.002		.031
Psychological Distress	r	.487^**^	-.359^**^	.060	-.176^*^	1
	P	.000	.000	.464	.031	
Sleep Quality	r	-.190^*^	-.015	.208^*^	.049	-.190^*^
	P	.020	.856	.011	.551	.020

## Discussion

This study examined the relationship between patients' overall ICU experience, psychological distress, and sleep quality among Jordanian burn patients. Based on the current study's findings, most patients were female and young, consistent with previous studies conducted in Jordan [14-15]. One of the significant findings is that most burn patients in the current study reported poor sleep quality. Previous studies reported that poor sleep quality was mainly associated with several factors, such as burn injury's physiological and psychological impacts [16]. As well as the ICU environment is also considered one of the primary sources of poor sleep and insomnia [17]. The ICU environment plays a significant role in sleep quality alteration among burn patients [18], adding further stress and impacting those patients. As this study was conducted among patients with burns in ICU settings, this could help in understanding the reported poor sleeping quality among burn survivors.

Burn survivors frequently deal with long-term physical, psychological, and environmental problems and suffer from numerous psychological and social stressors [19]. Psychological problems such as sadness, anxiety, and post-traumatic stress are reported by about 50% of burn survivors [20]. In our study, burn survivors reported severe psychological distress, which aligned with the literature findings. These stressors have been reported to influence the sleep quality among burn patients; for that, those patients commonly report several psychological disorders such as anxiety and depression [21-22].

From another angle, contractures, joint deformities, neurologic and musculoskeletal issues, scars, discomfort, itch, temperature intolerance, and exhaustion are a few examples of physical difficulties [2]. As these complications increase, patients' psychological distress also increases. Congruently with the literature, the burn patients in our study who had burn complications were more psychologically distressed than others. Furthermore, the current study showed that burn survivors had reported less satisfaction with care despite their ICU experience awareness. These findings could be related to several factors, including but not limited to the nature of the ICU environment and interruptions. Prior research has also emphasized the significance of burn patient satisfaction with care since it can affect treatment compliance and foster patient participation in their care [23].

In the current study, length of ICU stays, sleep quality, and psychological distress were associated with burn survivors' ICU experience after burn injury. Following a burn injury, several studies have shown a connection between psychological distress and more prolonged hospital admissions; more prolonged hospitalizations indicate longer wound healing times, which could be why patients experience overall negative experience with ICU care [24]. The current study revealed that the patients who spent more days in ICU reported more psychological distress. A recent study supported this finding, as Spronk et al. (2018) found that prolonged stay is associated with more psychological distress [19].

From social contexts, burn patients have reported several emotional barriers, such as the fear of rejection, feelings of self-consciousness, shame, and humiliation [25-27, 28]. Thinking about such ideas could affect the patients' feelings of frightening. Our study highlighted a contradicting finding as patients spent more days in ICU. They had more psychological distress but less frightening experience. The rationale for that could rely on the community and social system structure in Jordan and the Middle East. In Arab culture, the connectedness with the social system appears as the relationship between patients, family members, neighbors, and surrounding contexts [29]. The social system encourages relatives and neighbors to visit ill patients. In our culture, most family members must visit the patient at home or hospital to support them. For this reason, despite having psychological distress, our patients' fear of the ICU decreased.

## Conclusions

Based on the study's findings, we gave evidence that burn injury significantly leads to poor sleep quality and psychological distress after ICU admission. The burn cases are taken into consideration here. ICU hospitalized burn patients show that the length of ICU stay was associated with psychological distress and frightening experience. Also, severe burn injuries can interfere with sleep state and psychological distress. In the future, we recommend that healthcare providers regularly evaluate the sleep of all burn patients, regardless of how they had recall and awareness experience after the accident. When screening and treating sleep disturbances in burn populations, it may be crucial to consider the psychosocial factors during ICU admission. It has been noted that assessing patients’ psychological distress and sleep quality should become a part of usual ICU care.
